# Plastid Deficient 1 Is Essential for the Accumulation of Plastid-Encoded RNA Polymerase Core Subunit β and Chloroplast Development in *Arabidopsis*

**DOI:** 10.3390/ijms222413648

**Published:** 2021-12-20

**Authors:** Zhipan Yang, Mingxin Liu, Shunhua Ding, Yi Zhang, Huixia Yang, Xiaogang Wen, Wei Chi, Congming Lu, Qingtao Lu

**Affiliations:** 1 Key Laboratory of Photobiology, Institute of Botany, Chinese Academy of Sciences, Beijing 100093, China; yangzp@ibcas.ac.cn (Z.Y.); liumingxin@ibcas.ac.cn (M.L.); dshhua@ibcas.ac.cn (S.D.); hxyang@ibcas.ac.cn (H.Y.); wenxg@ibcas.ac.cn (X.W.); chiweimr@ibcas.ac.cn (W.C.); 2 College of Life Science, University of Chinese Academy of Sciences, Beijing 100049, China; 3 State Key Laboratory of Crop Biology, College of Life Sciences, Shandong Agricultural University, Taian 271018, China; zhangyi@sdau.edu.cn

**Keywords:** *Arabidopsis*, PD1, PEP, chloroplast development, chloroplast gene expression

## Abstract

Plastid-encoded RNA polymerase (PEP)-dependent transcription is an essential process for chloroplast development and plant growth. It is a complex event that is regulated by numerous nuclear-encoded proteins. In order to elucidate the complex regulation mechanism of PEP activity, identification and characterization of PEP activity regulation factors are needed. Here, we characterize Plastid Deficient 1 (PD1) as a novel regulator for PEP-dependent gene expression and chloroplast development in *Arabidopsis*. The *PD1* gene encodes a protein that is conserved in photoautotrophic organisms. The *Arabidopsis* *pd1* mutant showed albino and seedling-lethal phenotypes. The plastid development in the *pd1* mutant was arrested. The PD1 protein localized in the chloroplasts, and it colocalized with nucleoid protein TRXz. RT-quantitative real-time PCR, northern blot, and run-on analyses indicated that the PEP-dependent transcription in the *pd1* mutant was dramatically impaired, whereas the nuclear-encoded RNA polymerase-dependent transcription was up-regulated. The yeast two-hybrid assays and coimmunoprecipitation experiments showed that the PD1 protein interacts with PEP core subunit β (PEP-β), which has been verified to be essential for chloroplast development. The immunoblot analysis indicated that the accumulation of PEP-β was barely detected in the *pd1* mutant, whereas the accumulation of the other essential components of the PEP complex, such as core subunits α and β′, were not affected in the *pd1* mutant. These observations suggested that the PD1 protein is essential for the accumulation of PEP-β and chloroplast development in *Arabidopsis*, potentially by direct interaction with PEP-β.

## 1. Introduction

Chloroplasts are unique plant organelles that are the site of photosynthesis and some critical metabolic pathways [1,2,3]. As a remnant of cyanobacterial endosymbiosis, chloroplasts keep their own genome and gene expression systems. The proper expression of the chloroplast genome is essential for the biogenesis and the development of chloroplasts [4]. The chloroplast genome of higher plants contains 120~210 thousand base pairs, encoding rRNA, tRNA, and approximately 70~80 protein-encoding genes [5,6]. They are transcribed by nuclear-encoded RNA polymerase (NEP) and/or plastid-encoded RNA polymerase (PEP) [7,8,9]. NEP, a single-subunit polymerase, is responsible for the expression of housekeeping genes, such as *RNA polymerase subunit* (*rpo*) genes, as well as several genes involved in gene expression and other essential plastid functions. PEP is a multi-subunit polymerase that is composed of five core subunits (2α, β, β′, and β″) encoded by the plastid *rpoA*, *rpoB*, *rpoC1*, and *rpoC2* genes [9,10]. PEP is responsible for driving the high-level transcription of the photosynthesis-related genes in the chloroplast genome and is necessary for generating functional chloroplasts [11,12]. The Δ*rpo* plants with deletion of the PEP core subunit show pigment-deficient and lethal phenotypes. The plastids in Δ*rpo* plants are arrested in the proplastid stage and lack the arrays of stacked thylakoid membranes for active photosynthesis [13,14]. 

The PEP core complex, with only five core subunits, has been biochemically purified from etioplasts or intermediate greening chloroplasts [15,16]. In fully developed chloroplasts, the PEP core enzyme is associated with additional subunits of eukaryotic origin. Twelve PEP-associated proteins (PAPs) that exhibit a strong and reproducible interaction with the core complex were identified by biochemical purification of chloroplast PEP enzymes followed by mass spectrometry identification. They were taken as necessary components of functional chloroplast PEP [17,18]. Genetic studies have demonstrated that *Arabidopsis* knockout lines for each *PAP* gene show an albino or pale-green phenotype with severe defects in the chloroplast development and the PEP activity [4,17,18]. The PAPs possess a wide range of diverse functions including DNA/RNA metabolism (PAP1/pTAC3, PAP2/pTAC2, PAP3/pTAC10, PAP5/pTAC12, PAP7/pTAC14, PAP8/pTAC6, and PAP12/pTAC7), redox-dependent regulation (PAP6/FLN1 and PAP10/TRXz), and reactive oxygen species scavenging (PAP4/FSD3 and PAP9/FSD2) [17,19], whereas none of the PAPs appear to possess a function that is truly essential for transcription [17]. Extensive interactions between PAPs have been reported [19,20,21,22,23,24,25]. It was proposed that the formation of the PEP complex through such PAP–PAP or PAP–PEP interactions may be the bottleneck governing PEP activity and chloroplast development [18]. 

In addition to the PAPs mentioned above, some other vital regulation factors of the PEP activity located in the nucleoid have been reported recently. Several of them participate in the gene expression of PEP core subunits, such as pentratricopeptide repeat (PPR) family protein CLB19, OTP70, YS1, and OsPPR16, which are involved in the editing or splicing of *rpo* genes [26,27,28,29], as well as the mitochondrial transcription termination factor (mTERF) family protein mTERF6, which is involved in the transcription termination of *rpoA* polycistron [30]. Some nucleoid proteins affect PEP activity by interacting with PAPs. FLN2 (FRUCTOKINASE-LIKE 2) was identified in the pTACs complex and interacted with PAP proteins PAP6/FLN1 and PAP10/TRXz. However, unlike *fln1* mutant, the *fln2* mutant only shows the chlorosis and slow-greening phenotypes [24,31]. One thioredoxin-like fold protein, MRL7/AtECB1, may play a role in thioredoxin signaling to regulate PEP-dependent plastid gene expression. It has thioredoxin activity and interacts with both PAP10/TRXz and PAP4/FSD3 [32,33]. FTRc, the catalytic subunit of Fd:TRX reductase, interacts with PAP10/TRXz, and the corresponding mutant shows a dramatically reduced PEP-dependent gene expression and impaired chloroplast development. It is most likely that FTRc and TRXz act together to regulate PEP function during the early stages of the chloroplast development [34]. PRIN2 (Plastid Redox Insensitive 2), another protein that interacts with PAP10/TRXz, is required for the full PEP activity in the chloroplasts. The monomerization of PRIN2 is probably mediated by PAP10/TRXz and provides a mechanistic link between photosynthetic electron transport and the activation of photosynthetic gene expression [35,36]. In addition, nucleoid protein PRDA1 (PEP-Related Development Arrested 1) [37], DG238 (DELAYED GREENING 238) [38], and mTERF3 [39] are also functional in the regulating of PEP-dependent gene expression. They interact with PAP9/FSD2, PAP6/FLN1, and PAP5/7/12, respectively. These studies showed that PEP activity is highly regulated by a large number of protein factors. However, there are still some gaps between PEP activity and its regulation. Therefore, the identification and characterization of new regulation factors are needed to elucidate the PEP transcription regulation mechanism.

Here, we identified a novel factor, PD1, which is essential for chloroplast development. A series of analyses showed that PD1 is located in the chloroplast nucleoid and is required for PEP transcription activity. Moreover, PD1 most likely regulates PEP activity by interacting with PEP-β and affects the accumulation of PEP-β. Our study provides a new clue for understanding the complex regulating mechanism of PEP activity.

## 2. Results

### 2.1. pd1 Mutant Shows the Albino and Seedling-Lethal Phenotypes

**Figure 1:**
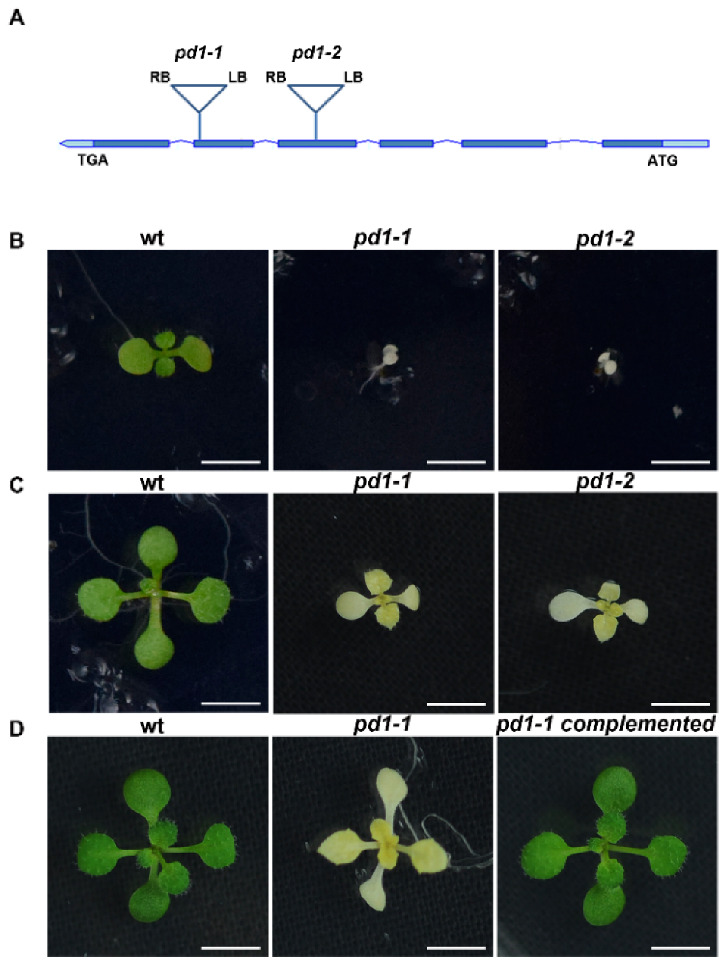

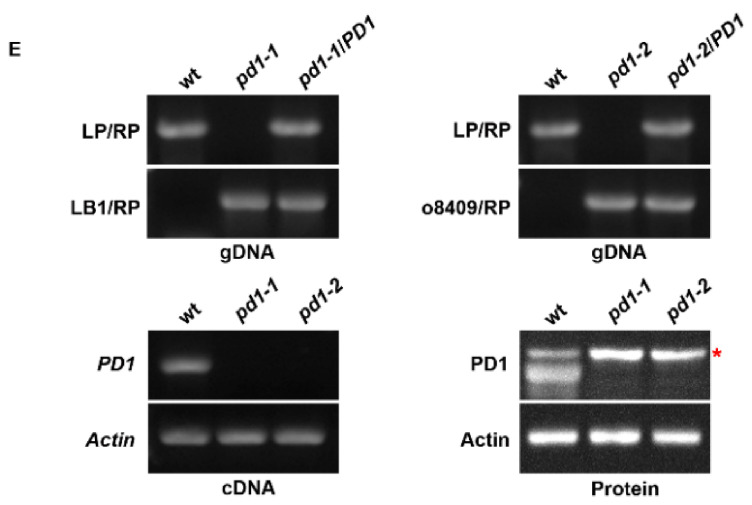
*pd1* mutants show albino and seedling-lethal phenotypes. (**A**) Schematic diagram showing *PD1* gene and the T-DNA insertion locus (bar topped by a triangle) of *pd1-1* and *pd1-2* mutants. (LB) Left border. (RB) Right border. (**B**) Two-week-old seedlings of wild type and *pd1* mutants grown in MS medium without sucrose. Bars = 1.0 cm. (**C**) Two-week-old seedlings of wild type and *pd1* mutants grown in MS medium with 3% sucrose. Bars = 1.0 cm. (**D**) Three-week-old seedlings of wild type, *pd1-1* mutant and complemented plants grown in MS medium with 3% sucrose. Bars = 1.0 cm. (**E**) Identification of *pd1* mutants at DNA, RNA, and protein levels. LP and RP, upstream and downstream primers for *PD1* gene; LB1 and o8409, primers of T-DNA insertion for different collections. The asterisk marks a nonspecific band.

In order to find new regulators of chloroplast development, we screened a large number of mutants of predicted chloroplast protein. One mutant line, CS825665, segregated albino seedlings, indicating that the chloroplast development of this mutant may be defective. We named it *pd1-1* (*plastid deficient 1-1*). The genome analysis showed that there was a T-DNA insertion in the fifth exon of the *At4g37920* gene, which resulted in a severe albino phenotype and retarded growth of *pd1-1* in MS medium without sucrose (Figure 1A,B,E). Another mutant line (GK347964, *pd1-2*), with an insertion in the fourth exon of *At4g37920*, showed a similar phenotype (Figure 1A,B,E). When cultured in MS medium with 3% sucrose, the homozygote of *pd1-1* and *pd1-2* could sustain survival and growth to an extent, but still failed to accumulate the pigments (Figure 1C, Table 1). When the seedlings were transplanted into soil, the *pd1* mutants stopped growing and died gradually. As a result, the seeds of the *pd1* mutants could only be harvested from heterozygotes. As *pd1-1* and *pd1-2* had the same phenotype, the following *pd1* mutants were represented by *pd1-1*.

In order to ensure that the albino phenotype of *pd1* was induced by the knockout of the *At4g37920* gene, the CDS sequence of *At4g37920* driven by the *CaMV 35S* promoter was constructed into a *pCAMBIA1301* vector and was transformed into heterozygotes of *pd1-1*. After the antibiotic screening and genome analysis of the T1 generation of seedlings, it was confirmed that the defective *PD1* is responsible for the phenotype of the *pd1* mutant (Figure 1D). 

### 2.2. pd1 Mutant Shows Defects in Chloroplast Development and Thylakoid Biogenesis

**Figure 2:**
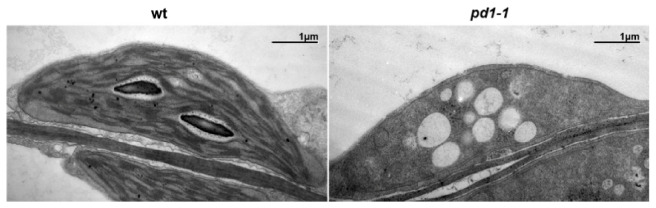
Transmission electron microscopic images of chloroplasts in leaves from two-week-old wild type and *pd1-1* mutant seedlings grown on MS medium with 3% sucrose. Bars = 1 μm.

In order to investigate the effect of the defective PD1 on chloroplast development, we used transmission electron microscopy to examine the morphology and ultrastructure of the plastids in the true leaves of the two-week-old wild type and the mutant plants grown in MS medium with 3% sucrose. Under normal growth conditions, the chloroplasts in the wild type leaves showed a lens shape and had well-organized thylakoid membrane systems composed of stroma and grana thylakoids (Figure 2). However, the chloroplasts in the *pd1-1* mutant leaves had no thylakoid structure and only had some oval-shaped vesicle structures (Figure 2). These results indicate that the plastid development and thylakoid biogenesis in *pd1* were severely arrested. 

**Figure 3:**
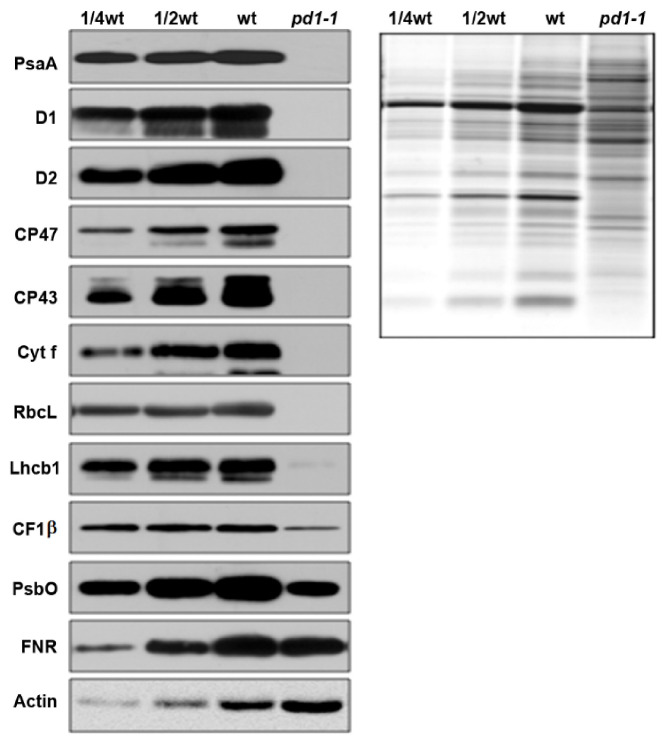
Immunoblot analysis of photosynthetic proteins in wild type and *pd1-1* seedlings. Immunoblot analysis of photosynthetic proteins based on the equal total leaf proteins was performed using two-week-old wild type and *pd1-1* seedlings that were grown on MS medium with 3% sucrose. Total leaf proteins were extracted and separated by SDS-urea-PAGE. The immune detection was probed with specific antibodies.

Following the developmental status of the chloroplasts in *pd1-1*, the accumulation of photosynthetic machinery proteins in the mutant decreased dramatically compared to those of the wild type (Figure 3). The core components of PSII (D1, D2, CP43, and CP47), PSI (PsaA), Cytb6f (Cyt f), and Rubisco (RbcL) were barely detected in the *pd1* mutants. The levels of nuclear-encoded PSII components, such as Lhcb1, PsbO, and the contents of the proteins related to electron transfer, such as FNR and the β-subunit of ATP synthase (CF1β), also declined to different extents. These results suggest that PD1 plays an important role in the accumulation of chloroplast photosynthetic proteins.

### 2.3. PD1 Gene Encodes a Novel Protein Conserved in Various Photoautotrophic Organisms

**Figure 4:**
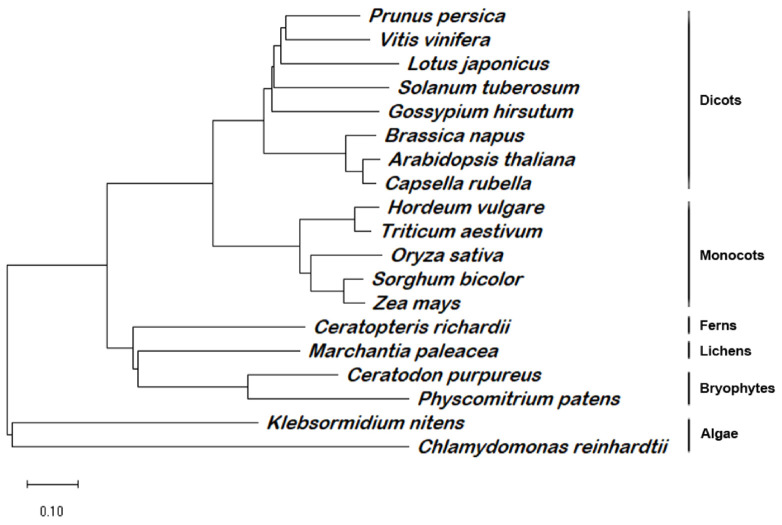
Phylogenetic analysis of PD1 homologous proteins. Full-length homologous amino acid sequences of PD1 protein in *Arabidopsis thaliana* (AT4G37920.1), *Prunus persica* (XP_007205240.1), *Vitis vinifera* (CBI25713.3), *Solanum tuberosum* (XP_006357761.1), *Gossypium hirsutum* (XP_040934732.1), *Lotus japonicus* (AFK48093.1), *Brassica napus* (CAF2146351.1), *Capsella rubella* (XP_006282520.2), *Oryza sativa* (NP_001042846.1), *Hordeum vulgare* (BAK00948.1), *Triticum aestivum* (KAF7071860), *Sorghum bicolor* (XP_002437093.1), *Zea mays* (NP_001144269.1), *Ceratopteris richardii* (KAH7301390.1), *Marchantia paleacea* (KAG6541617.1), *Ceratodon purpureus* (KAG0589879.1), *Physcomitrium patens* (XP_024372527.1), *Klebsormidium nitens* (GAQ78741.1), and *Chlamydomonas reinhardtii* (XP_042914248.1) were selected to generate a bootstrap Neighbor-joining phylogenetic unrooted tree by MEGA 11. Subclades representing evolutionary linkages are marked. Scale bar = 0.1 amino acid substitutions.

PD1/AT4G37920 was annotated in public databases (e.g., TAIR and NCBI) as a chloroplast protein without any known conserved domain. The sequence-alignment analysis revealed the presence of PD1 orthologs in many photosynthetic organisms, including green algae, bryophytes, lichens, ferns, and angiosperms. However, we did not find the orthologs from the gymnosperm. In order to better present the evolutionary relationship among PD1 proteins, we constructed a phylogenetic tree using MEGA 11 [40]. The PD1 proteins showed a clear evolutionary linkage from lower green algae to various higher plants (Figure 4). Furthermore, the PD1 proteins from the angiosperms formed the main subclade with two subclades of dicots and monocots. The PD1 proteins from green algae, bryophytes, lichens, and ferns formed the other four subclades. These results suggest that PD1 is conserved in various photoautotrophic organisms. There could be two possible reasons for the absence of the gymnosperm PD1 orthologs. First, there is no PD1 ortholog sequence information for the lack of complete genome annotation information in gymnosperms. Second, PD1 orthologs were lost in the gymnosperm genome during the evolution process.

### 2.4. PD1 Gene Mainly Expresses in Green Tissues 

**Figure 5:**
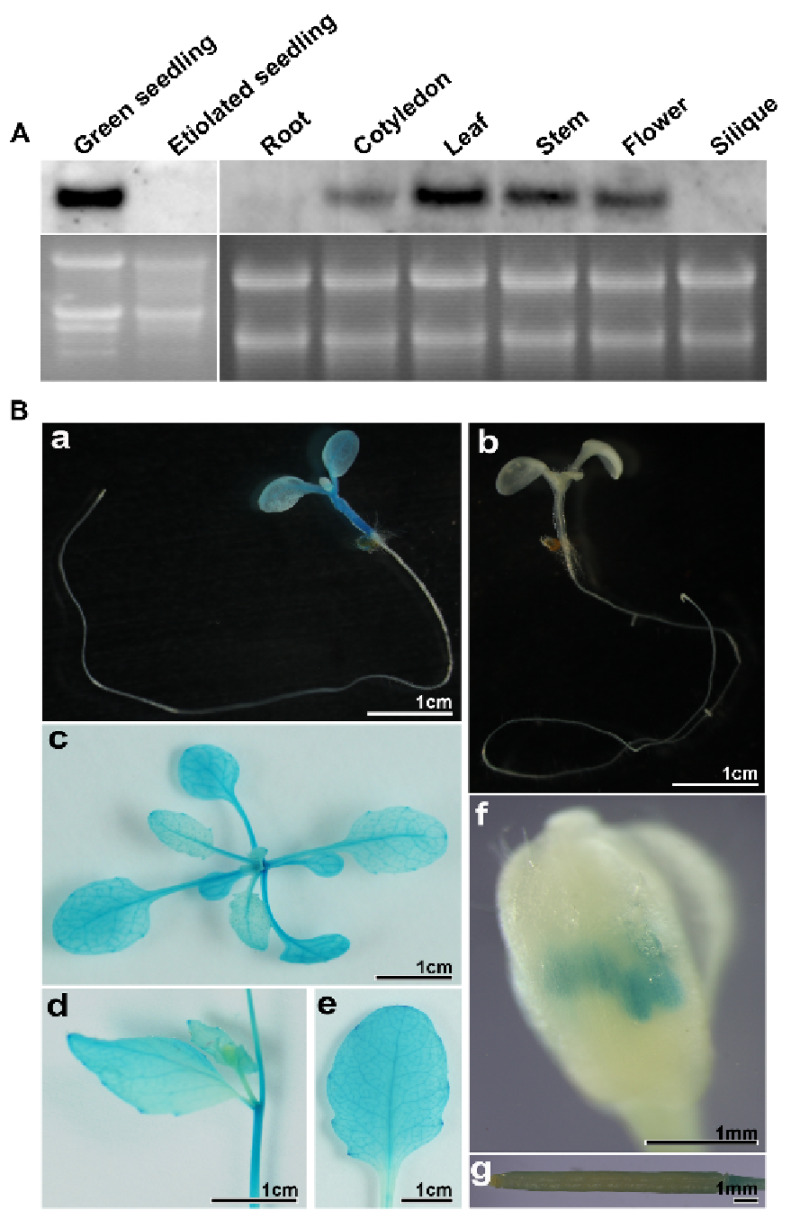
Expression characteristics of the *PD1* gene. (**A**) RNA accumulation of the *PD1* gene in different tissues and developmental stages. Total RNA was extracted from seven-day-old green seedlings and etiolated seedlings, roots, cotyledons, leaves, stems, flowers, and siliques and underwent formaldehyde electrophoresis and northern blot analyses. 10 μg total RNA per lane. (**B**) GUS staining of transgenic plants expressing *GUS* gene driven by *PD1* promoter. (**a**) ten-day-old transgenic seedling expressing *GUS* gene driven by *PD1* promoter. (**b**) ten-day-old seedling transformed *pCAMBIA1381Z* empty vector. (**c**–**g**) rosette leaf, stem, cauline leaf, flower, and silique from the transgenic plants expressing *GUS* gene driven by *PD1* promoter.

There is little spatio-temporal expression information about the *PD1/At4g37920* gene in the public databases. In order to learn more about its spatio-temporal expression patterns, we determined the level of *PD1* mRNA in the different tissues and developmental stages of the wild type plants by northern blot analysis. No *PD1* mRNA was detected in the etiolated seedlings. A high *PD1* mRNA level was observed in the cotyledons, rosette leaves, stems, and flowers. The *PD1* mRNA was barely expressed in the roots and siliques (Figure 5A). In order to further investigate the spatio-temporal expression information of *PD1*, we generated transgenic plant lines expressing the *β-glucuronidase* (*GUS*) reporter gene driven by the *PD1* promoter. The results of GUS staining showed that *PD1* was expressed in the cotyledons, rosette leaves, stems, cauline leaves, and flowers, but was not expressed in the roots and siliques. The *PD1* gene was expressed exclusively in the anthers of the flowers but not in the petals and pistils (Figure 5B). These results indicate that the *PD1* gene is mainly expressed in the green tissues.

### 2.5. PD1 Is Localized in the Chloroplast Nucleoid

**Figure 6:**
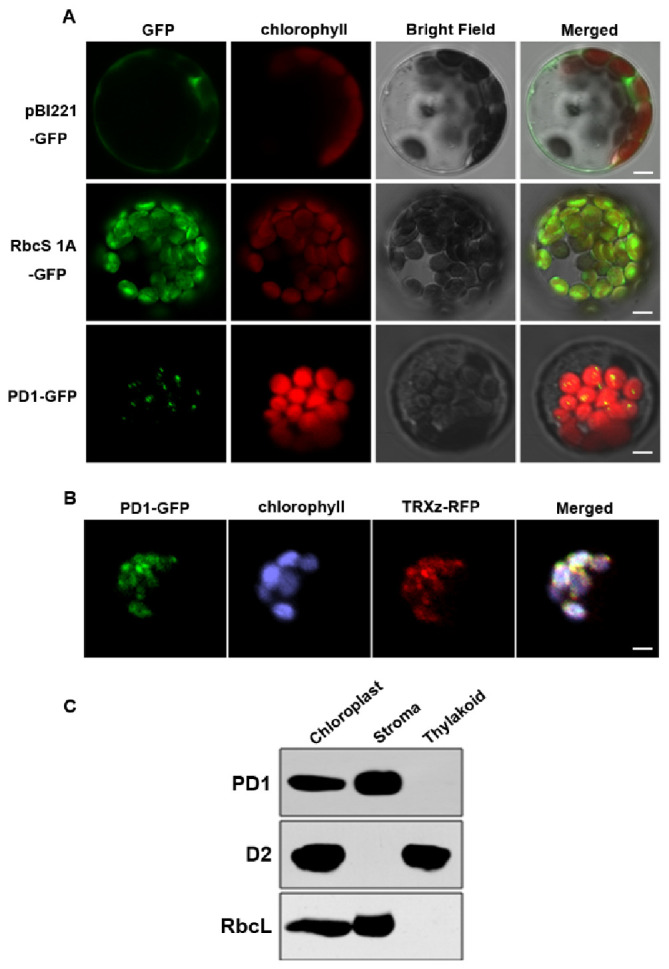
Subcellular localization of the PD1 protein. (**A**) Localization of PD1 protein within chloroplast by GFP assay. Chimeric proteins were transiently expressed in *Arabidopsis* protoplasts. pBI221-GFP, control with the *pBI221* empty vector; RbcS 1A-GFP, chloroplast control; PD1-GFP, PD1-GFP fusion. Chlorophyll autofluorescence of chloroplasts is shown in red. Bars = 3 μm. (**B**) Colocalization of PD1-GFP with TRXz-RFP. The fluorescence signal of PD1-GFP overlaps with that of TRXz-RFP within chloroplast nucleoids. Chlorophyll autofluorescence of chloroplasts is shown in purple. Bars = 3 μm. (**C**) PD1 localizes in the chloroplast stroma. Intact chloroplasts were isolated from the leaves of wild type seedlings and then separated into thylakoid membrane and stroma fractions. Polyclonal antisera against the PD1, D2, and RbcL were used in immunoblot analysis.

PD1 was annotated in public databases (TAIR, www.arabidopsis.org, accessed on 12 May 2017) and was predicted (TargetP-2.0, https://services.healthtech.dtu.dk/service.php?TargetP-2.0, accessed on 12 May 2017) to encode an unknown chloroplast protein. In order to investigate the subcellular localization of PD1, PD1-green fluorescent protein (GFP) fusion was introduced into the *Arabidopsis* protoplasts, and GFP fluorescence was found to be localized to the chloroplasts (Figure 6A). As the GFP fluorescence was in a dotted pattern, it is possible that PD1 is associated with the nucleoids. We further examined whether PD1-GFP was colocalized with red fluorescent protein (RFP) fused with TRXz, a well-characterized protein localized in nucleoids [21]. The fluorescence signal overlay of PD1-GFP and TRXz-RFP indicated that PD1 and TRXz colocalized in the chloroplast nucleoids (Figure 6B). To further investigate the sub-chloroplast localization of the PD1 protein, intact chloroplasts extracted from the two-week-old wild type seedlings were fractionated to the stroma and the thylakoid membrane, for which RbcL and D2 were selected as the marker proteins, respectively. The immunoblot analysis showed that the PD1, similarly to RbcL, was detected only in the stroma fraction (Figure 6C).

### 2.6. pd1 Mutants Have Defects in PEP-Dependent Plastid Gene Transcription

**Figure 7:**
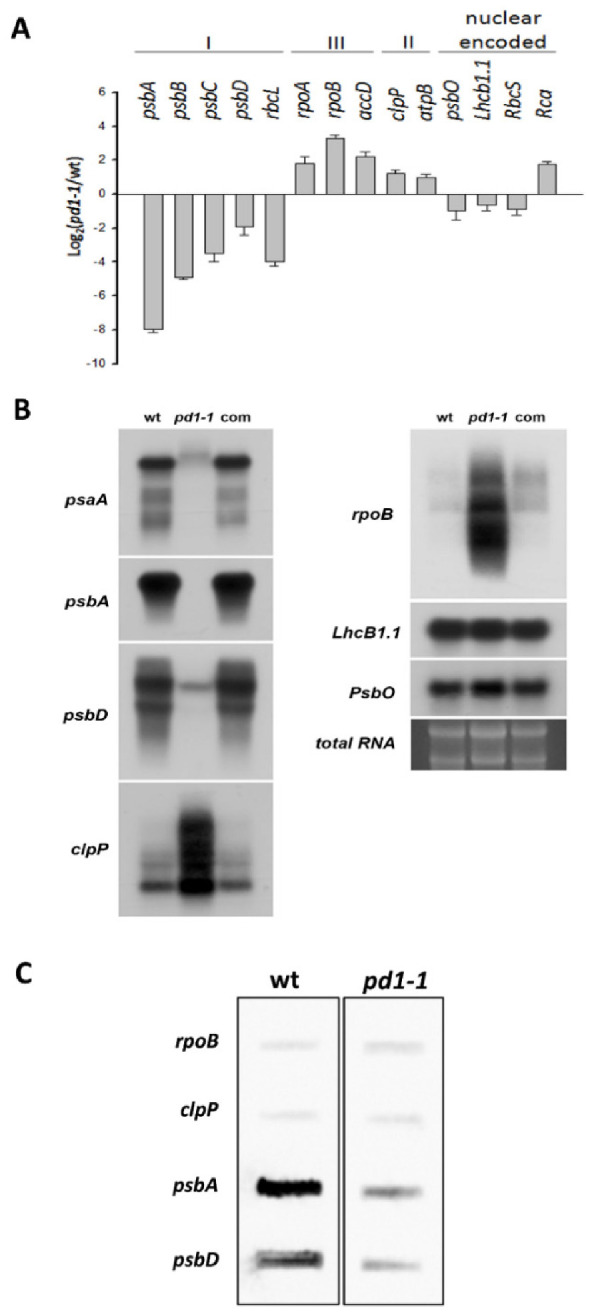
Transcriptional analysis of the chloroplast genes in wild type, *pd1-1*, and complemented seedlings. (**A**) Analysis of the chloroplast gene transcripts by RT-quantitative real-time PCR. The log_2_ (*pd1-1*/wt, *pd1-1* mutant/wild type) values were calculated and normalized by using *actin* as a reference. I. PEP-dependent chloroplast genes; II. NEP-dependent chloroplast genes; III. Both PEP- and NEP-dependent chloroplast genes. The values shown are the average of three independent replicates. Error bars indicate SD. (**B**) Analysis of chloroplast gene transcripts by northern blot. The RNA on the agarose gel stained with ethidium bromide is shown as a loading control. 10 μg total RNA per lane. (**C**) Analysis of the transcriptional rate of the chloroplast genes in wild type and *pd1-1* plants via run-on assay. The filters were probed with run-on transcripts derived from chloroplasts isolated from wild type and *pd1-1* seedlings. The experiments were repeated three times independently, and the data from one representative experiment are presented.

Since PD1 was located in the chloroplast nucleoids, it is likely to be involved in chloroplast gene expression. To confirm this, the relative expression levels of plastid genes in the wild type and the *pd1* mutants were studied by RT-quantitative real-time PCR and northern blot analysis. Chloroplast genes are divided into three classes according to RNA polymerase-dependence. Class I is PEP-dependent, class II is both NEP- and PEP-dependent and class III is NEP-dependent. Our results showed that in the *pd1* mutant, the transcript levels of the class I genes (*psbA*, *psbB*, *psbC*, *psbD*, and *RbcL*) were down-regulated consistently and the transcript levels of class II (*clpP* and *atpB*) and class III genes (*accD*, *rpoA*, and *rpoB*) were up-regulated (Figure 7A,B). The expression of nuclear-encoded genes (*Lhcb1.1*, *Rca*, *RbcS*, and *PsbO*) that encode proteins targeted to chloroplasts displayed slight changes in the *pd1* mutants (Figure 7A,B). 

In order to determine whether the decrease in the mRNA levels of the PEP-dependent genes is induced by the impaired PEP activity in *pd1* mutants, we performed run-on assays with isolated chloroplasts from the two-week-old *Arabidopsis* seedlings. As shown in Figure 6C, the transcription rate of *psbA* and *psbD* declined obviously in the mutant, whereas those of *rpoB* and *clpP* were up-regulated slightly compared with the wild type. These results indicated that PEP transcription activity decreased markedly in *pd1* mutants.

### 2.7. PD1 Interacts with PEP-β

**Figure 8:**
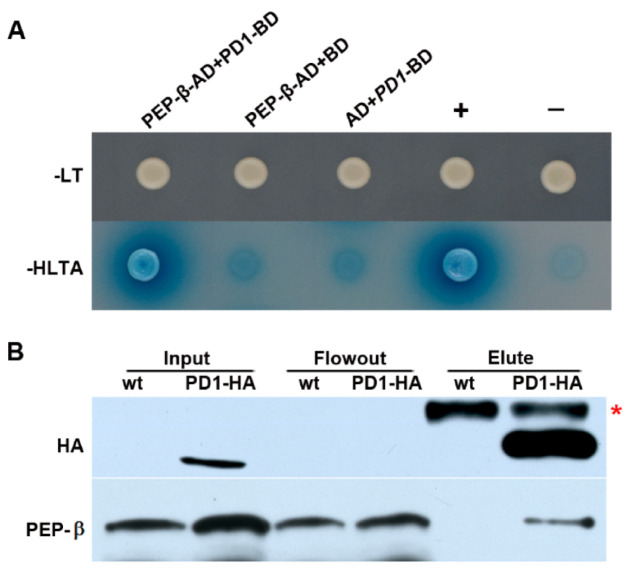
PD1 interacts with PEP-β. (**A**) Yeast two-hybrid assays. The predicted mature PD1 protein fused to the GAL4 DNA-binding domain (BD) was expressed in combination with PEP-β proteins fused to the GAL4 activation domain (AD) in yeast strain AH109. Cells were grown on different selective media. Empty BD and AD vectors served as negative controls. -LT, SD medium without Leu and Trp; -HLTA, SD medium lacking His, Leu, Trp, and Ade. X-α-Gal was added to -HLTA plate to indicate the expression of the *MEL1* reporter gene. +, Positive control expressing *pGADT7-T* and *pGBKT7-53*; −, Negative control expressing *pGADT7-T* and *pGBKT7-Lam*. (**B**) Coimmunoprecipitation assay. Total leaf protein extracted from two-week-old wild type and transgenic seedlings expressing PD1-HA tag fusion protein received CoIP assay by employing Anti-HA-tag mAb-Magnetic Beads. The samples of input, flowout, and elute were examined by specific antibodies for HA and PEP-β, respectively. The asterisk shows a nonspecific band.

The function of PD1 is unknown. Searching for the interaction factors of PD1 is a good way to explore its function. To this end, the predicted mature PD1 protein, ranging from amino acid 63 to 427, was used in fusion with the GAL4 BD as bait to screen the two-hybrid cDNA libraries from *Arabidopsis*. Of the ~2 × 10^7^ primary transformants, 96 clones were selected as being positive in growing on selective plates and were analyzed by sequencing. Finally, 26 putative chloroplast proteins, encoded by 34 inserts, were identified. Among these potential interaction proteins, PEP-β encoded by *rpoB* is directly related to PEP transcription. The two inserts encoding the *rpoB* gene consisted of the same sequence starting with residue 866. The specificity of the interaction between PEP-β and PD1 was verified by a yeast two-hybrid experiment with PEP-β-AD and PD1-BD (Figure 8A).

In order to test the interaction between PD1 and PEP-β in *Arabidopsis*, coimmunoprecipitation assay was carried out by using the transgenic seedlings expressing the PD1-HA tag fusion protein. The analysis suggested that PD1 interacts with PEP-β in vivo (Figure 8B).

### 2.8. Accumulation of PEP-β Is Defective in pd1 Mutant 

**Figure 9:**
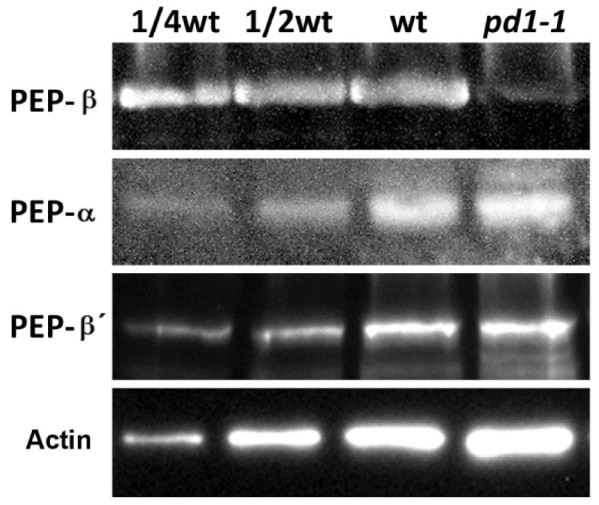
Accumulation of PEP-β subunit is defective in the *pd1-1* mutant. Total leaf protein prepared from wild type and *pd1-1* seedlings was separated by SDS–PAGE electrophoresis and analyzed by immunoblot with specific antibodies for PEP-β. Accumulation of PEP-α and PEP-β′ was examined parallelly. Accumulation of Actin was the reference of the loading sample.

In order to understand the effect of PD1 on PEP-β, we examined the protein accumulation of PEP-β in the *pd1* mutant. The immunoblot analysis indicated that the accumulation of PEP-β was barely detectable in the *pd1* mutant. The accumulation of PEP core subunits α and β′ did not change (Figure 9). These results suggested that PD1 is essential for the accumulation of PEP core subunit β.

## 3. Discussion

The plastid gene expression during chloroplast development can be divided into three steps. In the first step, plastid DNA synthesis and replication are activated. In the second step, the genes encoding the plastid gene expression machinery are transcribed by nuclear-encoded RNA polymerase (NEP) for the rapid establishment of the plastid genetic system [9]. In the final step, the relative activity of NEP is reduced and maintained at the basal level. In contrast, the plastid-encoded RNA polymerase (PEP) activity increases and is maintained at a high level, which leads to the strong expression of plastid-encoded components of the photosynthetic apparatus [41]. Thus, the generation of the full PEP complement is an essential step during chloroplast development [18]. It is known that many chloroplast development mutants are defective in the PEP structure or function; they usually show pigment deficiency and seedling-lethal phenotypes [13,14,17,18]. The *pd1* mutants showed seedling-lethal phenotypes accompanied with white cotyledons under autotrophic conditions (Figure 1B). On medium supplemented sucrose, the primary leaves of the *pd1* mutants showed ivory phenotypes and the mutants could sustain survival and growth to an extent, which implied that the plastids were still active [17]. However, the chlorophyll content in the *pd1* mutants was still very low compared to the wild type (Figure 1C, Table 1). This suggests that the photosynthesis apparatus and/or function of the *pd1* mutants were impaired severely. In the *pd1* mutant, the ultrastructure of the chloroplasts had no thylakoid membrane structure, which was replaced by oval-shaped vesicles (Figure 2). This was also observed in Δ*rpo* [14] and some *pap* mutants, such as *pap5*/*ptac12* [42], *pap7*/*ptac14* [22], *pap8*/*ptac6* [42], and *pap12*/*ptac7* [25]. Therefore, the *pd1* mutants are very similar to *pap* and Δ*rpo* mutants regarding their visible phenotypes, as well as their chloroplast ultrastructure. These results suggest that PD1 plays an essential function in the early stage of chloroplast development. In accordance with its crucial role in chloroplast development, the *PD1* gene is expressed mainly in the green tissues, but not in etiolated seedlings (Figure 5), and the *PD1* gene is localized in the chloroplasts (Figure 6A). Furthermore, PD1 exists in most photoautotrophic organisms, from green algae to angiosperms (Figure 4). This means that, as with some other important PEP regulator factors, such as PAP7/pTAC14 [22], PRDA1 [37], and DG238 [38], the emergence of PD1 is synchronous with the occurrence of chloroplasts. 

The chloroplast nucleoids are the central location of plastid DNA/RNA metabolism, ribosome assembly, and many other processes associated with chloroplast development [43]. Previous research on the maize nucleoid proteome indicated that the homologous protein of PD1 could be identified in the maize nucleoid [43]. In this study, the subcellular localization analyses of the PD1-GFP fusion proteins revealed that they were colocalized with the characterized nucleoid protein TRXz (Figure 6B). These results suggested that PD1 is a chloroplast nucleoid-associated protein. It is possible that PD1 is involved in chloroplast gene expression and regulation. The analysis of plastid gene expression revealed that, in *pd1* mutants, the transcript levels of chloroplast genes were dramatically changed (Figure 7). The PEP-dependent chloroplast transcripts were barely detected in the *pd1* mutants, whereas those of the NEP-dependent chloroplast transcripts were increased (Figure 7A,B). These are typical features of the Δ*rpo* mutants and have been observed in many PEP-deficient mutants [17]. The run-on assay further indicated that the transcription rate in the *pd1* mutants decreased dramatically compared to that in the wild type (Figure 7C). These observations suggested that PD1 is essential for the maintenance of PEP function. The accumulation levels of some important chloroplast proteins, such as the core components of PSII (D1, D2, CP43, and CP47), PSI (PsaA), and Rubisco (RbcL), were barely detected in the *pd1* mutants (Figure 3). These proteins were encoded by the PEP-dependent genes, and impaired PEP activity should be an important reason for their decreased accumulation. 

In recent years, many nucleoid proteins have been found to be related to the PEP activity in the chloroplast, including 12 PAPs and other regulation factors of PEP transcription [17,31,32,33,34,35,36,37,38,39]. Despite their functional importance, the molecular mechanism of these PEP regulation factors in plastid transcription is largely unknown. Extensive interactions between PAPs, and PAP and other PEP regulators have been observed. For instance, PAP3/pTAC10 interacts with PAP9/FSD2, PAP4/FSD3, PAP10/TRXz, PAP12/pTAC7, and PAP7/pTAC14 [44]; PAP4/FSD3 interacts with PAP9/FSD2 [20]; PAP7/pTAC14 interacts with PAP5/pTAC12 [22]; PAP10/TRXz interacts with PAP6/FLN1 and FLN2 [21,24]; PAP12/pTAC7 binds to PAP3/pTAC10, PAP5/pTAC12, PAP7/pTAC14, and PAP6/FLN1 [25]; mTERF3 interacts with PAP5/pTAC12, PAP7/pTAC14, and PAP12/pTAC7 [29], and MRL7 interacts with PAP10/TRXz and PAP4/FSD3 [32]. However, none of them have been reported to directly interact with PEP core subunits. Although the association of PAP1/pTAC3 and PEP-α with transcribed regions in vivo was examined by using chloroplast chromatin immunoprecipitation assays [23], there is still a lack of direct evidence of their interaction. Here, we identified that PD1 directly interacts with PEP-β using the yeast two-hybrid and coimmunoprecipitation methods (Figure 8A,B). This suggests that PD1 is a novel PEP regulation factor, and it may have different regulation mechanisms from the previously identified regulation factors of PEP.

PEP-β is the core subunit of the PEP complex. It has been reported that missing PEP-β could result in the albino and seedling-lethal phenotypes, as well as the arrested chloroplast development and a significant decrease in PEP activity [13,14]. In the *pd1* mutant, we observed that the accumulation of PEP-β was barely detectable (Figure 9), which may induce the Δ*rpo* phenotype of the *pd1* mutants. A lower accumulation of PEP-β was also observed in the rice *osppr16* mutant, which has impaired editing at position 545 in the chloroplast *rpoB* messenger RNA and the mutant shows a pale phenotype at the seedling stage [29]. In *Arabidopsis*, a lack of editing at *rpoB*-338 in *ys1* mutants led to a virescent phenotype at the early leaf development stage [28]. The *Arabidopsis crr22* mutants, which were impaired in the editing of *rpoB-551* (corresponding to *rpoB-545* in rice), *ndhB-746*, and *ndhD-887* did not show any obvious phenotype under the standard growth conditions [45]. However, the accumulation levels of PEP-β in the *ys1* and *crr22* mutants were not reported [28,45]. OsPPR16, YS1, and CRR22 are all pentratricopeptide repeat (PPR) family proteins, which are defined by the tandem array of a PPR motif consisting of 35 amino acids. Most PPR proteins are predicted to be located in the plastid or the mitochondria, playing essential roles in the posttranscriptional regulation of organelle gene expression [46,47]. Whereas PD1 has no conserved domain, the editing efficiency of *rpoB* in the *pd1* mutants was not changed compared to the wild type (data not shown). Therefore, this suggests that the lower accumulation of PEP-β in the *pd1* mutants is not related to *rpoB* editing. Considering that PD1 interacts with PEP-β directly, we suggest that PD1 might play a role in maintaining the stability of PEP-β. The details of the molecular mechanism of PD1 regulating PEP-β accumulation remains to be studied.

In conclusion, PD1 is an essential regulator of chloroplast development and PEP activity; it directly interacts with PEP-β and is essential for the accumulation of PEP core subunit β. To date, PD1 is the first factor for maintaining the stability of PEP-β. In addition, PD1 is a conserved protein in photoautotrophic plants, and the orthologs of PD1 were found in many important crops. Therefore, our research on PD1 provides not only novel insight into PEP activity regulation, but also provides potential helpful targets for chloroplast engineering and crop yield improvement. 

## 4. Materials and Methods

### 4.1. Plant Material and Growth Conditions

The *Arabidopsis thaliana* (ecotype Columbia) wild type and T-DNA insertion line *pd1-1* and *pd1-2* were obtained from ABRC and the GABI-KAT collection. *Arabidopsis* seeds were surface-sterilized and sown on Murashige and Skoog (MS) medium with and without 3% (*w*/*v*) sucrose and 0.4% (*w*/*v*) gellan gum. After being cold-treated for two days, they were grown at 22 °C under short-day conditions (12 h of light/12 h of dark) with a photon flux density of 100 μmol m^−2^ s^−1^. After two weeks of growth on plates, the seedlings were harvested for experiments or transferred to soil and cultured in a growth chamber under the same conditions. The pigment was extracted from two-week-old seedlings with 80% acetone and analyzed according to the method described by Lichtenhaler [48].

### 4.2. Protein Alignment and Phylogenetic Analyses

The sequences of PD1 and its homologous proteins from representative species were obtained from TAIR (www.arabidopsis.org (accessed on 20 October 2021)) and by BLASTP alignments with e-values of less than 1E-10 in GenBank (blast.ncbi.nlm.gov (accessed on 20 October 2021)). The multiple sequence alignments and phylogenetic analyses were performed with MEGA 11 software [40].

### 4.3. Plant Transformation and Complementation Analysis

Full-length CDS of *PD1* gene fusion with HA-tag was cloned into the *pCAMBIA1301* vector with the cauliflower mosaic virus *35S* promoter. The constructs were then transformed into *Agrobacterium tumefaciens* strain GV3101pMP90 and introduced into *PD1*/*pd1* heterozygous mutant plants using the floral dip method [49]. T1 generation plants were screened on MS medium containing 40 mg/L hygromycin. Resistant plants with *pd1*/*pd1* homozygous backgrounds were selected by genome analysis employing the PCR method with specific primers (Table S1).

### 4.4. Histochemical Staining

The *GUS* reporter gene driven by the *PD1* promoter (1183 bp upstream of predicted *PD1* transcriptional initiation point) was cloned into *pCAMBIA1381Z* vector and transferred into *Arabidopsis* Columbia ecotype. Transgenic positive seedlings were selected by hygromycin and used for GUS staining. More than ten independent transgenic lines were acquired, and three of them received histochemical analysis. Plant samples were incubated in GUS staining solution overnight at 37 °C in the dark. Then, they were treated with 70% ethanol several times to remove chlorophyll. After clearing, the samples were observed and photographed employing an SZX16 stereomicroscope (Olympus, Center Valley, PA, USA) and digital camera (D3300, Nikon, Tokyo, Japan). Fresh GUS staining solution was prepared as follows: 0.1 M sodium phosphate buffer (pH 7.0), 0.5 mM K_3_[Fe(CN)_6_], 0.5 mM K_4_[Fe(CN)_6_], 10 mM Na_2_EDTA, 0.1% (*v*/*v*) Triton X-100, and 1 mg/mL X-Gluc.

### 4.5. Transmission Electron Microscopy

Transmission electron microscopy was performed according to our previous study [50]. Leaves from two-week-old seedlings grown in MS medium with 3% sucrose were collected. The samples were observed with a transmission electron microscope (JEM-1230; JEOL, Tokyo, Japan).

### 4.6. Subcellular Localization of GFP Proteins

Full-length CDS of the *PD1* and *TRXz* were cloned into *pBI221*-GFP and *pBI221*-RFP vectors. The resulting fusion constructions and the control vectors were transformed into protoplasts of *Arabidopsis*. Fluorescence was observed by confocal scanning microscopy (LSM 510 Meta, Zeiss, Oberkochen, Germany). A 488 nm laser line and 505 to 530 nm band pass filter were used for GFP excitation and emission, respectively. RFP was excited with the 543 nm laser line and detected using the 600 to 630 nm band pass filter. Chlorophyll auto-fluorescence was excited with the 488 nm laser line and was detected using a 650 nm band pass filter.

### 4.7. SDS-PAGE and Immunoblot Analysis

Total proteins were extracted for immunoblot analysis as described by Martinez-Garcia et al. [51]. Total leaf proteins were separated using 15% SDS polyacrylamide gels containing 6 M urea. After electrophoresis, the proteins were transferred to PVDF microporous membrane (IPVH00010, Merck, Darmstadt, Germany). The membranes were incubated with specific primary antibodies and the signals from secondary conjugated antibodies were detected using the enhanced chemiluminescence method. PsaA, D1, D2, CP43, CP47, Cyt f, CF1β, RbcL, and FNR antibodies were produced in our laboratory [52]. PEP-α, PEP-β, PEP-β′, PsbO, Lhcb1.1, and Actin antibodies were purchased from PhytoAB Inc. (San Jose, CA, USA).

### 4.8. PD1 Antiserum Production

For the production of polyclonal antibodies against PD1, the nucleotide sequences encoding the mature PD1 (amino acids 63-427) were amplified by RT-PCR. The resulting DNA fragment was cleaved with *EcoR* I and *Xho* I and fused in a frame with the N-terminal His affinity tag of *pET28a* (Novagen, Madison, WI, USA), and the resulting plasmid was transformed into *Escherichia coli* strain BL21 (DE3). The fusion protein was purified on a nickel–nitrilotriacetic acid agarose resin matrix and raised in a rabbit with purified antigen.

### 4.9. RT-PCR, RT-Quantitative Real-Time PCR and Northern Blot Analysis

Total RNA was isolated from plant leaves employing TRIzol (Invitrogen, Carlsbad, CA, USA) and used for cDNA synthesis and northern blot analysis. The cDNA was synthesized using a PrimeScript^TM^ II 1st Strand cDNA Synthesis Kit (6210A, Takara, Kusatsu, Japan) with RQ1 RNase-free DNase (M610A, Promega, Madison, WI, USA). RT-PCR and RT-quantitative real-time PCR analyses were performed according to a previous study [53]. The amplification of *actin* was used as an internal control for normalization. For an overview of the primers used, see Table S1. For northern blot analysis, total RNAs were fractionated with formaldehyde denaturing 1.2% agarose gel and transferred onto nylon membranes (RPN303B, GE Healthcare, Buckinghamshire, UK) as described by Sambrook and Russell [54]. The membranes were probed with ^32^P-labeled probes that were prepared as described by Yu et al. [55].

### 4.10. Chloroplast Run-On Assay

The chloroplast run-on assays were carried out as described by Chi et al. [56]. Plant samples of wild type and *pd1* mutant were prepared from two-week-old seedlings and labeled with DIG-11-UTP (11209256910, Roche, Rotkreuz, Switzerland) according to the manual from Roche Applied Science. DNA probes (300 ng) of *rpoB*, *clpP*, *psbA* and *psbD* were amplified by RT-PCR and blotted onto Hybond N+ nylon membranes by a slot blotting apparatus (Bio-DotSF, Bio-Rad, Hercules, CA, USA). Hybridization was carried out employing a hybridization oven (HL-2000 HybriLinker^TM^, UVP) overnight in 6× SSC, 5× Denhardt’s solution, 0.5% SDS and 50 μg/mL yeast tRNA at 65 °C. Hybridization results were analyzed by the exposure and development of X-ray film.

### 4.11. Coimmunoprecipitation (CoIP) Assay

The CoIP experiment was carried out employing transgenic seedlings expressing the PD1-HA tag fusion protein and Anti-HA-tag mAb-Magnetic Beads (M180-11, MBL, Sunnyvale, CA, USA) in strict accordance with the product instructions. The samples underwent immunoblot examination by using antibodies for PEP-β and HA-tag purchased from PhytoAB Inc (San Jose, CA, USA), respectively.

### 4.12. Yeast Two-Hybrid Assay

The screening of the Mate & Plate Library-Universal *Arabidopsis* (Normalized) (630487, Clontech, Mountain View, CA, USA) and the yeast two-hybrid assay were performed using the Matchmaker GAL4 Two-hybrid System according to the user manual (PT4084-1, Clontech, Mountain View, CA, USA). The *PD1* coding sequence (free of its predicted chloroplast transit peptide) fused to *pGBKT7* containing a DNA-binding domain (BD) constituted the bait plasmid, and the candidate proteins fused to *pGADT7* containing a DNA activation domain (AD) constituted the prey plasmid. The bait plasmid and prey plasmid were co-transformed into yeast reporter strain AH109. These transformants were grown on SD/-Trp-Leu and SD/-Trp-Leu-His-Ade dropout selective culture medium. The interactions between the bait and prey proteins were verified by testing the *MEL1* gene activity.

## Figures and Tables

**Table 1:** Chlorophyll and carotenoid contents in leaves of wild type and *pd1* mutants.

Line	Chlorophyll *a* (μg g^−1^ FW)	Chlorophyll *b* (μg g^−1^ FW)	Total Chlorophyll (μg g^−1^ FW)	Carotenoids (μg g^−1^ FW)
wild type	1123.81 ± 17.13	409.74 ± 13.49	1533.55 ± 24.26	50.60 ± 1.19
*pd1-1*	5.50 ± 1.05	6.23 ± 0.98	11.73 ± 2.01	3.53 ± 0.11

Mean ± SD values were calculated from three independent experiments based on fresh weights (FW).

## Data Availability

The data presented in this study are available in this article and Supplementary Material.

## Supplementary Materials

The following are available online at https://www.mdpi.com/article/10.3390/ijms222413648/s1.
